# Effects of carcass decomposition on vegetation dynamics in Bankenveld grasslands of South Africa

**DOI:** 10.1098/rsos.250509

**Published:** 2025-10-08

**Authors:** Resoketswe Cate Khumalo, Alan Barrett, Leslie R. Brown

**Affiliations:** ^1^ Environmental Science, University of South Africa College of Agriculture and Environmental Sciences, Roodepoort, Gauteng, South Africa; ^2^ Environmental Science, University of South Africa College of Agriculture and Environmental Sciences, Pretoria, Gauteng, South Africa; ^3^ Applied Behavioural Ecology and Ecosystems Research Unit, University of South Africa, Pretoria, Gauteng, South Africa

**Keywords:** Telperion Nature Reserve, plant species composition, nutrient cycling, veld condition, carrion, biomass production

## Abstract

Carcass decomposition plays a crucial role in nutrient cycling and ecosystem functioning, yet its impact on vegetation dynamics in different ecosystems remains poorly understood. This study investigates the effects of decomposing adult blue wildebeest (*Connochaetes taurinus*) carcasses on vegetation dynamics in the Bankenveld grasslands of South Africa, a biodiverse and ecologically significant ecosystem. Field experiments conducted at Telperion Nature Reserve examined changes to vegetation structure, biomass production, plant species composition and nutritive value associated with the decomposition of blue wildebeest carcasses with three treatment types (control/no carcass, open carcass and caged carcass) over a period of 12 months. Results show an increase in vegetation height at all carcass sites, an increase in plant cover at caged carcass sites, a significant increase in biomass at control and caged carcass sites, a significant increase in species richness at carcass sites and no change in species diversity with a smaller decrease in veld condition scores at caged carcass sites showing a slight improvement in veld condition. These findings show that decomposition noticeably affected the variables investigated, promoting increased growth of nutrient-demanding species and altering community composition. This study suggests that carcass decomposition contributes towards the heterogeneity of Bankenveld grasslands, emphasizing that decomposition processes should be considered in management and conservation strategies for these areas.

## 1. Introduction

Carcass decomposition is a complex process involving the breakdown of animal remains and the release of nutrients into the surrounding environment [1]. This process plays an important role in nutrient cycling, energy transfer and the maintenance of ecosystem health [2,3]. Nutrients released from a carcass affect the local soil chemistry and fertility [1,4]. Carcass decomposition alters soil fertility beneath and immediately around the site for many years, influencing nutrient availability and microbial diversity [5,6].

To date, numerous studies have investigated the effects of animal carcass decomposition on arthropod successional patterns [3,7,8], nutrients released by decomposing carcasses [9–11] and soil dynamics related to decomposing carcasses [9,12]. However, very few studies have investigated the impacts of carcass decomposition on spatial and temporal vegetation dynamics [9,13].

The impact of large herbivore carcasses (animals with a body weight greater than 5 kg) on plant nutrient concentration has been studied in various locations [14], including Kenyan groundwater wooded grassland [15], North American tall-grass prairie [5], Canadian arctic tundra [6], Australian box gum grassy woodland [12] and in Bialowieza Primeval Forest [4]. These studies have employed various methodological approaches to understand decomposition effects, with exclusion cages proving particularly valuable for separating the effects of nutrient inputs from those of scavenger activity. Exclusion cages allow researchers to control for the confounding effects of large and small scavengers, which can significantly alter carcass decomposition rates and nutrient distribution patterns [16]. By comparing caged and uncaged carcasses, researchers can better isolate the direct effects of decomposition on vegetation while accounting for the indirect effects of scavenger-mediated nutrient dispersal and soil disturbance. This experimental approach has been instrumental in demonstrating that scavenger exclusion typically leads to more concentrated nutrient patches and different temporal patterns of vegetation response compared with naturally scavenged carcasses [17].

To date, there are no recorded studies of the impacts of large herbivore carcass decomposition on grassland vegetation in southern Africa. In nutrient-poor ecosystems, large carcasses provide small periodic resource islands, which influence vegetation structure and composition years after an animal’s death [12]. Grassland ecosystems are areas dominated by grasses and other herbaceous plants [18]. The grassland biome covers 29% of South Africa and has the second highest biodiversity compared with other South African biomes [19,20]. Bankenveld grassland, specifically, is a topographically heterogeneous landscape providing habitats for many rare, endangered and endemic plant and animal species [20–22]. These grasslands provide crucial ecosystem services, including carbon storage, water filtration and habitat for numerous plant and animal species. However, Bankenveld is facing increasing threats from land-use changes, such as agricultural expansion and climate change [23]. Understanding the dynamics of key ecological processes, such as carcass decomposition, is essential for developing effective conservation and management strategies in this region.

The aim of this study was to determine the impacts of adult blue wildebeest (*Connochaetes taurinus*) carcass decomposition on vegetation structure, biomass production, plant species composition and nutritive value in the Bankenveld grassland of South Africa. To achieve this aim, we employed a controlled field experiment using three treatment types: control sites (no carcass), open carcass sites (exposed to natural scavenging) and caged carcass sites (protected from large scavengers). This experimental design allowed us to distinguish between the direct effects of nutrient inputs from decomposition and the indirect effects of scavenger activity on vegetation dynamics. Data were collected over a period of 12 months to capture both immediate and longer-term responses to carcass decomposition. The objectives were to determine the effect of decomposition on: (i) biomass production; (ii) vegetation structure/height; (iii) spatial and temporal changes to plant species composition and cover; and (iv) nutritive value (veld condition) of grasses along a spatial gradient from the carcass site outwards, as well as over time.

We hypothesized that blue wildebeest decomposition within the study site would lead to an increase in plant biomass production, vegetation height, composition, cover and veld condition 1 year post-decomposition. Specifically, we predicted that: (i) caged carcass sites would show greater increases in biomass production and vegetation height compared with open carcass sites due to more concentrated nutrient inputs; (ii) both carcass treatments would exhibit higher plant species richness and altered species composition compared with control sites, with a shift towards more nutrient-demanding species; (iii) plant cover would initially decrease at caged carcass sites due to vegetation mortality during early decomposition, but would subsequently increase above control and open carcass sites’ levels as enhanced nutrient availability stimulates growth; (iv) veld condition scores (VCS) would improve at carcass sites over time, reflecting increased grass vigour and palatability; and (v) spatial analysis would reveal that carcass-induced changes in vegetation height, biomass production, species composition and veld condition would be most pronounced within 2 m radius of carcass sites and would diminish with distance, creating distinct nutrient and vegetation gradients around decomposition sites.

## 2. Material and methods

### 2.1. Study area

**Figure 1.**
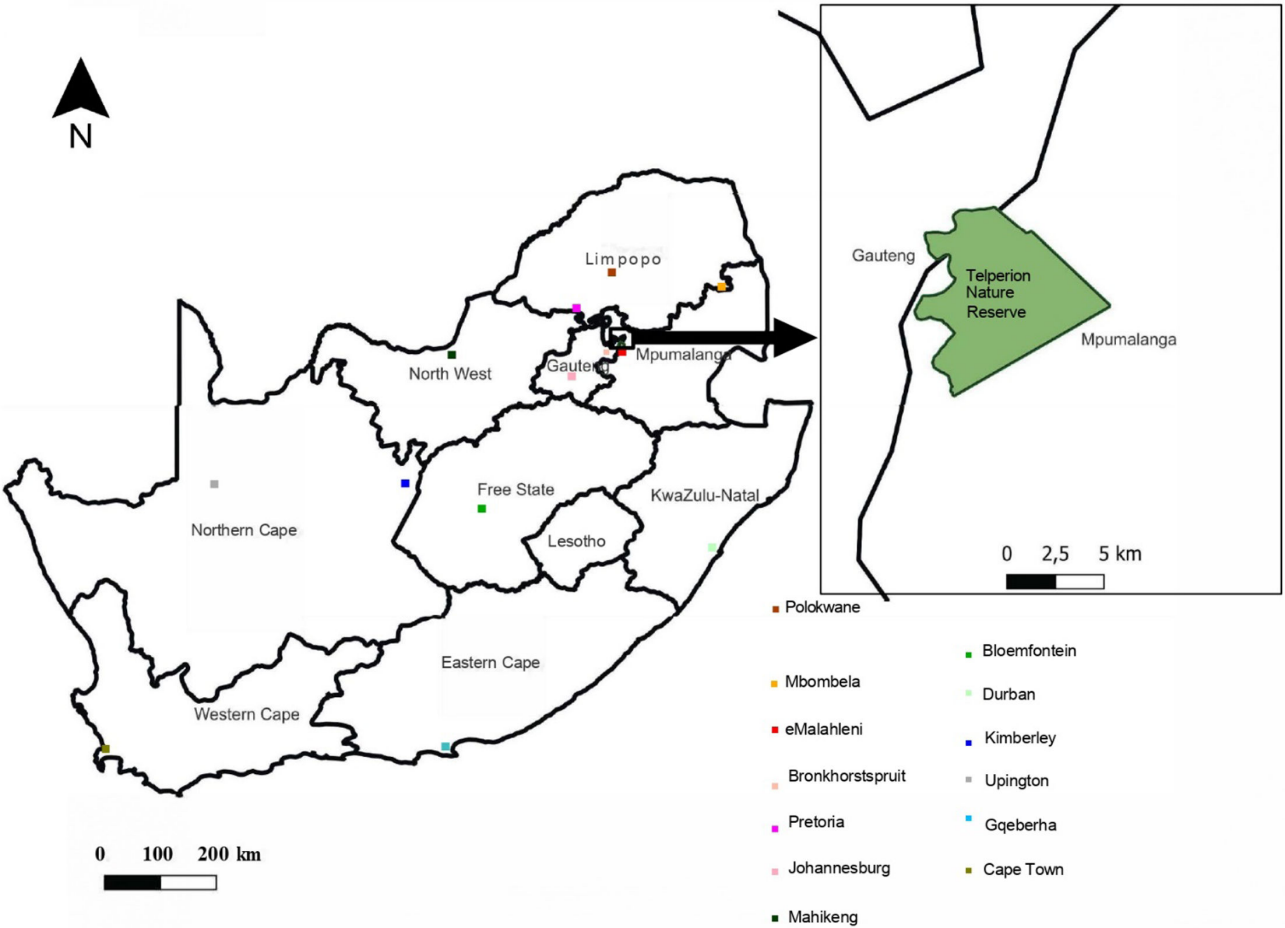
Location of Telperion Nature Reserve in South Africa.

Telperion Nature Reserve (TNR) is located approximately 25 km east of the town Bronkhorstspruit and 45 km west of the town eMalahleni in the Mpumalanga Province of South Africa (figure 1). The reserve is located on the eastern extremity of the Magaliesberg Mountain Range [24]. The coordinates for the reserve are 25°38′32.42″ S to 25°44′19.00″ S latitude and 28°58′41.98″ E to 29°04′2.06″ E longitude. Altitude ranges from 1200 m.a.s.l. at the lowest point to 1500 m.a.s.l. at the highest point [25]. The landscape is characterized by open, undulating grassy plains interspersed with wetland areas, and features rocky mountainous sections with plateaus in between. The western boundary is defined by the perennial Wilge River [26]. TNR, located in the summer rainfall region of South Africa, experiences warm, temperate and wet summers, and cool to cold dry winters [19]. The wet period is from November to April, and the dry period is from May to October [25]. Summer rainfall within TNR ranges from 570 to 730 mm per annum [19], with the highest rainfall recorded in January and the driest month being July [25].

Acocks [27] considers the area where the reserve is located to be a transition zone between the grassland and savannah biomes, occurring on high and low-lying inland plateaus. The vegetation of the grassland areas is classified as endangered Rand Highveld Grassland (vegetation type Gm 11), while the rocky hills and outcrops fall within the Loskop Mountain Bushveld (vegetation type SVcb 13) [19].

### 2.2. Sampling design

Ten senescent adult male blue wildebeest (*C. taurinus*) carcasses, harvested as part of the TNR annual culling operation, were used as a generic proxy for typical medium-to-large-sized African ungulate species. A total of 15 sample sites were chosen within the grassland vegetation of TNR. Three different carcass placement scenarios were established, each consisting of five sample sites. The scenarios included (i) five sites with carcasses pegged on wire platforms *in situ* (open carcass sites), allowing large- and medium-sized scavengers and predators to access and disperse carcass remains; (ii) five sites with carcasses enclosed within cages (caged carcass sites) to exclude large- and medium-sized scavengers and predators; and (iii) five control sites without carcasses. At the caged sites, carcasses were placed in custom built-to-purpose cages constructed from diamond mesh. For the open carcass sites, carcasses were pinned *in situ* to a built-to-purpose platform, also constructed from diamond mesh. Camera traps were employed to monitor scavenger activity as part of another study conducted simultaneously on animal behaviour at carcass sites, but not for this study. This is because the experimental design for this study provided clear evidence of scavenger presence and impact through the contrasting decomposition patterns and carcass remains between open and caged carcass sites. The rapid and complete consumption of soft tissues at open carcass sites, combined with the dispersal of skeletal remains, provided unambiguous evidence of scavenger activity, while the intact nature of caged carcasses confirmed successful exclusion of large vertebrate scavengers. Additionally, the focus of this study was on vegetation responses to decomposition rather than scavenger behaviour, making detailed documentation of individual scavenger species or activity patterns unnecessary for achieving the research objectives. Sample sites were spaced at least 1 km apart to promote spatial independence; however, we acknowledge that this distance may not be sufficient to ensure spatial independence for larger vertebrate scavengers. Sample site placement was based on vegetation structure to ensure all sites were in grassland areas with similar structural characteristics and broad species composition. Within these grassland areas, sample sites were randomly allocated to one of the three carcass placement scenarios.

**Figure 2.**
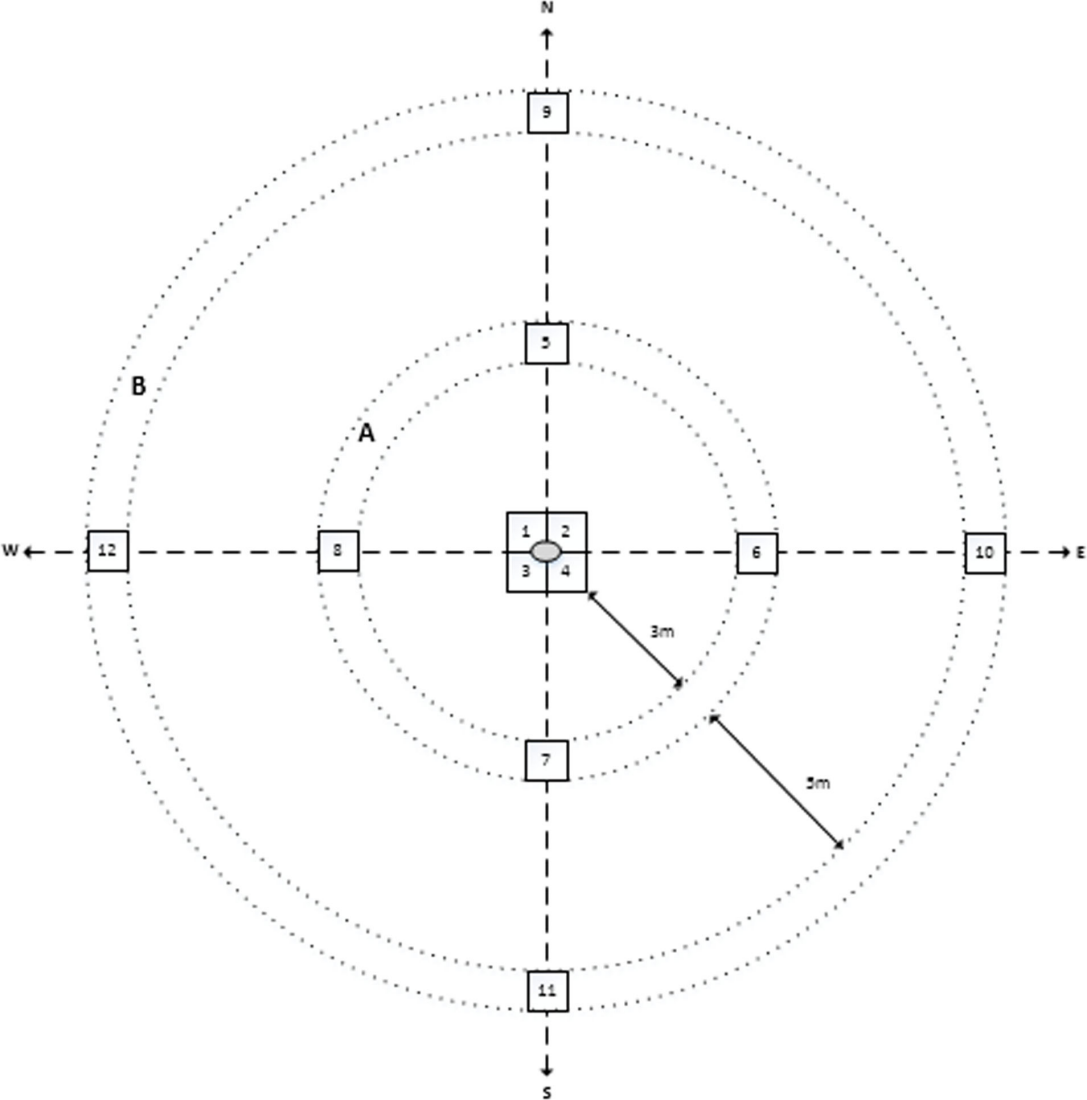
Layout of plant monitoring plots at each carcass and control site. The carcass is centrally located within plots 1 to 4 for sites with carcasses. Plots 5 to 8 are in ring A, starting 3 m away from the edge of the central plots. Plots 9 to 12 are in ring B, starting 5 m from the edge of the plots in ring A. All plots are 2 × 2 m in size, with each band measuring 2 m in width.

Baseline measurements were collected in January 2019 before the carcasses were placed in the field. Vegetation measurements were collected at each caged carcass site, open carcass site and control site. After baseline data collection, a second set of data was collected in April 2019, immediately following the complete decomposition of the carcasses, with only skin and skeletons remaining. A last set of vegetation data was collected in January 2020 for comparison with previously collected data. From the central points where each carcass was placed within each site, a central grid of four 2 × 2 m plots was established (figure 2).

From the central grid, four additional 2 × 2 m plots were placed in a band at 3 m from the end of the central plots, radiating out in the four cardinal directions. A second series of four 2 × 2 m plots, also radiating out in the four cardinal directions, was placed in a second band at 5 m from the edges of the plots in the first band (figure 2). A total of 12 sampling plots were placed at each site, resulting in 180 plots across 15 sites.

On the north and south-facing boundaries of each 2 × 2 m plot, five steel droppers were placed at 50 cm intervals starting from the eastern edge of the plot for data collection. Thus, there were 12 monitoring plots per site (figure 2). The four central plots will hereafter be referred to as the ‘centre’, with ‘Ring A’ referring to the four plots located 3 m from the edge of the central plots and ‘Ring B’ referring to the four plots located 5 m from the edge of the plots in Ring A (figure 2). All data were collected within these plots for each analysis. Data collected followed a series of transects within each plot. A rope was tied between each of the droppers, creating five parallel lines. At 10 cm intervals along these lines, a wooden pin was lowered, and the closest plant to the pin was noted.

Measurements collected in plots and at each data collection point in the plots included vegetation cover using the Domin scale [28], plant species name if any part of the plant was touched (or within 5 cm of the dropped wooden pin), and frequency/counts of species encountered. At each data collection point where the pin was dropped, the height class of the plant at or closest to the pin was recorded (table 1).

**Table 1.** Vegetation height ranges.

height class	height range
1	0 ≤ *h* ˂ 5 cm
2	5 ≤ *h* ˂ 10 cm
3	10 ≤ *h* ˂ 15 cm
4	15 ≤ *h* ˂ 20 cm
5	20 ≤ *h* ˂ 30 cm
6	30 ≤ *h* ≤ 50 cm
7	*h* ˃ 50 cm

A total of 100 plant biomass readings were taken within Ring A and Ring B, and 69 biomass readings were taken within the centre plots. Centre plots were restricted for space as these plots were connected to each other, hence the lower number of readings taken (figure 2). Biomass readings were taken by recording the height of a calibrated disc pasture meter (DPM) following the methodology described by Bransby & Tainton [29]. The DPM consists of a weighted aluminium disc (200 g, 46 cm diameter) that slides down a graduated rod and settles on the vegetation canopy, providing a height measurement that correlates with biomass. The disc was lowered gently onto the vegetation at each sampling point to avoid compressing the plant material, and the height reading was recorded where the bottom of the disc intersected the graduated rod. This height represents the compressed canopy height, which has been shown to correlate strongly with standing biomass in grassland systems [30]. The DPM was calibrated prior to the study using harvested biomass samples from similar grassland areas to establish the relationship between disc height readings and actual dry matter (DM) yield. At each sampling point, the DPM was positioned vertically above the vegetation, ensuring the disc remained level during descent to provide consistent readings. This procedure was repeated at systematic intervals across each plot, providing a standardized assessment of biomass that could be compared across treatments and over time.

Plant cover was determined by estimating the percentage of ground covered by all plants within each plot using a visual estimation technique. Within each 2 × 2 m plot, the observer estimated the percentage of ground surface covered by the vertical projection of all above-ground plant parts, including leaves, stems and reproductive structures. These plot estimates were then averaged to provide an overall plot cover percentage. To ensure consistency and reduce observer bias, cover estimates were made using standardized percentage classes: 0–5%, 6–25%, 26–50%, 51–75% and 76–100%, with the midpoint of each class used for calculations [31]. Where cover fell between classes, the observer assigned the estimate to the nearest 5% increment. All cover assessments were conducted by the same observer throughout the study period to maintain consistency in estimation techniques. Areas of bare soil, litter and rock were excluded from cover calculations, with only living plant material contributing to the cover estimate. Overlapping vegetation layers were accounted for by recording the maximum cover projection, meaning that if an upper canopy layer completely covered a lower layer, only the upper layer contributed to the total cover percentage at that location. This visual estimation method has been widely validated in grassland studies and provides data suitable for quantitatively determining changes over time from one collection event to the next [32].

Any plant species observed in a plot, but not sampled, was recorded, as well as the most common species observed. This approach ensured a comprehensive overview of the plant species in the area, capturing both less common species that might not have been sampled and the most common ones, which could play a significant role in the ecosystem.

For height measurement purposes, the wooden pin was marked at 5 cm intervals for height classes 1–4, as most vegetation in the plots fell within these classes (table 1). Height increments were then increased to 10 and 20 cm for height classes 5 and 6, respectively, as fewer plants exceed these heights. Height class 7 recorded plants taller than 50 cm (table 1). The height of the plant touched by the wooden pin, or the closest plant within a 5 cm radius, was measured and recorded according to the height classes specified in table 1 for consistency. Plants were measured from their bases to the highest plant part. This procedure was repeated at each 10 cm interval and along each parallel line along the rope, resulting in 100 height recordings per plot and 400 height recordings per centre, Ring A and Ring B plots (figure 2).

### 2.3. Statistical analysis

Vegetation cover, vegetation height and biomass data were recorded in Excel 2020 and analysed using one-way analysis of variance (ANOVA). To assess plant species composition and dynamics, both species richness and species diversity were analysed. Species richness was calculated as the total number of different species recorded in each 2 × 2 m sample plot, expressed as species per square metre. Species richness was simply the count of distinct species present in each plot, which is always an integer value (i.e. 1, 2, 3, etc. species).

Species diversity was assessed using the Shannon–Wiener diversity index (*H′*), which accounts for both species richness and evenness of species abundance across different functional groups such as forbs and grasses [33]. The Shannon–Wiener index was calculated using the following formula:


$$H^{\prime }=-\sum \left(\text{pi}\times \ln\;(\text{pi})\right),$$

where:

*H*' = Shannon–Wiener diversity index,

pi = proportion of individuals belonging to species *i* (calculated as ni/*N*, where ni is the number of individuals of species *i* and *N* is the total number of individuals), and

ln = natural logarithm.

The summation (Σ) extends from *i* = 1 to *s*, where *s* is the total number of species (species richness) recorded in the sample.

Since species richness (*s*) represents the actual count of species present in each plot, it is inherently an integer value, eliminating the need for rounding or truncation. The number of different species recorded in each sample area for the different treatment groups over time was calculated and organized using Microsoft Excel (2020).

The diversity values for each plant community were converted to effective species numbers as described by Jost [34], to facilitate meaningful comparisons between the different sample plots. The conversion to effective numbers was done using the following formula:


$$\text{Effective number of species}=\exp(H^{\prime })=\exp\left(-\sum (\text{pi}\times \ln(\text{pi}))\right),$$

where:

pi = proportion of individuals belonging to species *i* (consistent with the Shannon–Wiener calculation above), and

exp = exponential function (base *e*).

Box and whisker plots were produced using MS Excel (2020).

Veld condition scores were determined using the ecological index method (EIM), as described in Van Rooyen & Bothma [35]. Plant species were grouped into different ecological classes (Decreaser, Increaser I, Increaser II, Increaser III and Forb species) according to Van Oudtshoorn [36]. The number of species in each ecological class was calculated, summed for sites with the same treatments and year and categorized accordingly. Grouped bar charts were then produced using MS Excel (2020) to visualize total species counts by location, treatment and time, organized according to ecological class.

These values were further used to calculate VCS using the EIM. VCS were then used to assess changes in veld condition over time, particularly related to nutrient enrichment from decomposing carcasses. Statistical analyses were conducted using PAST [37], with ANOVA performed to determine significant differences in vegetation composition and VCS across treatments and sampling rings.

## 3. Results

### 3.1. Vegetation height

**Figure 3.**
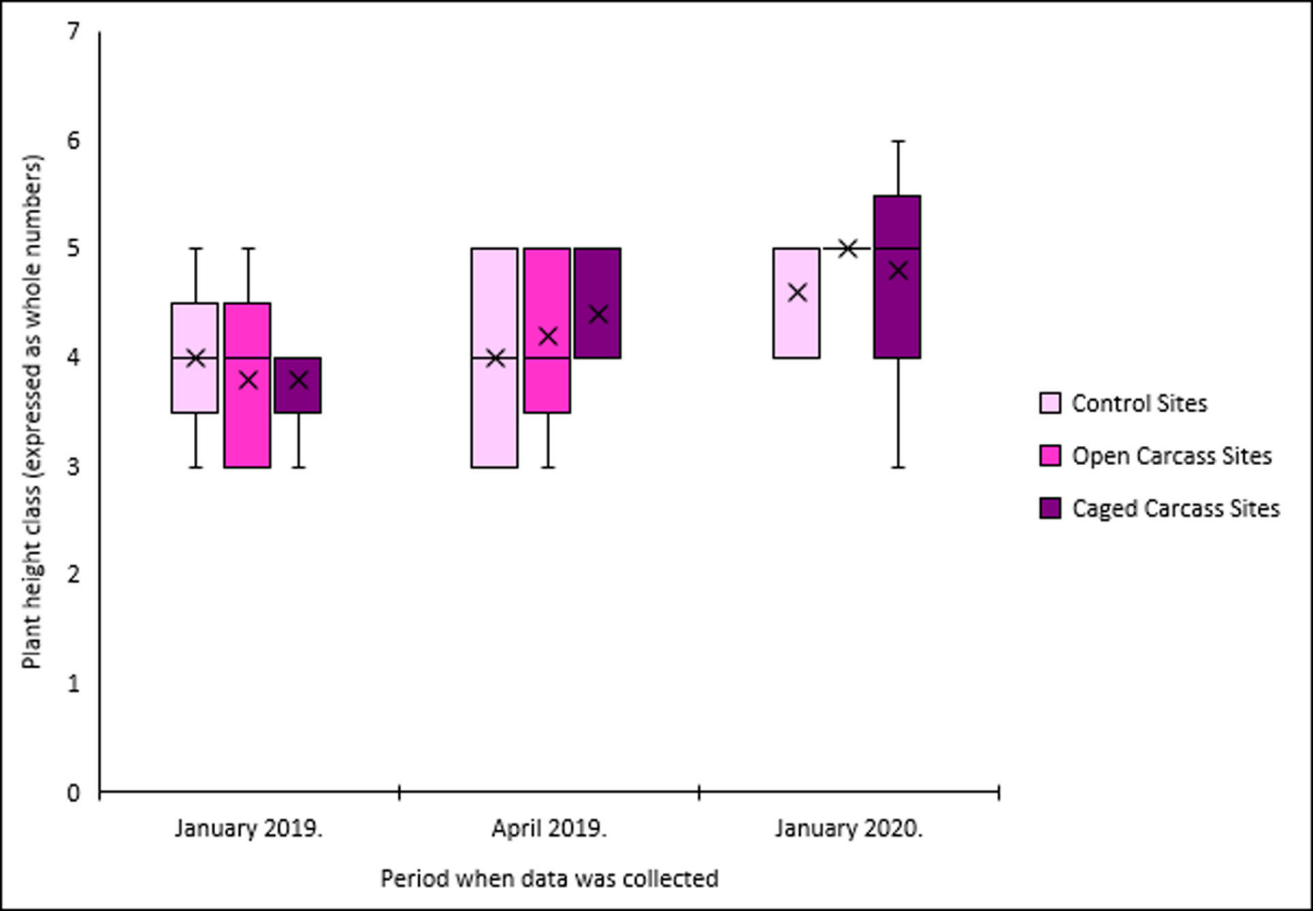
Vegetation height for all survey sites for the three different carcass placement scenarios over the study period.

Vegetation height increased at all carcass sites over the 12 months study period, while control sites showed minimal change (figure 3). Control sites maintained average heights of 15−20 cm throughout the study. Both open and caged carcass sites showed progressive increases from initial heights of 10−20 cm to final heights of 20−30 cm, though these increases were not statistically significant (table 2).

**Table 2.** Summary of results and predictions.

variable	prediction	result	statistical significance	prediction supported?
vegetation height	increased height at carcass sites versus controls	all carcass sites showed height increases; controls remained stable	not significant (open: *p* = 0.13; caged: *p* = 0.06)	partially—trend observed but not significant
plant cover	initial decrease, then increase above controls	caged carcass sites: 49% to 67% (+18%)	not significant (all *p* > 0.05)	partially—caged sites showed pattern
		open carcass sites: 60% to 61% (+1%)		
		control sites: 57% to 60% (+3%)		
species richness	higher richness at carcass sites	open carcass sites:+2 species	significant for carcass sites (open: *p* = 0.01; caged: *p* = 0.004)	yes
		caged carcass sites:+6 species		
		control sites:+2 species		
biomass production	greater biomass at caged > open > control	caged carcass sites:+15.9%	significant (all *p* < 0.001)	yes
		control sites:+10.58%		
		open carcass sites: −7.17%		
species diversity	increased diversity at carcass sites	no change across all treatments (approx. 2 effective species)	not significant (all *p* > 0.05)	no
veld condition	improved scores at carcass sites	all declined, but caged carcass sites declined least (control: −13%; open: −9%; caged: −7%)	not significant (all *p* > 0.05)	partially—relative improvement

### 3.2. Vegetation cover

**Figure 4.**
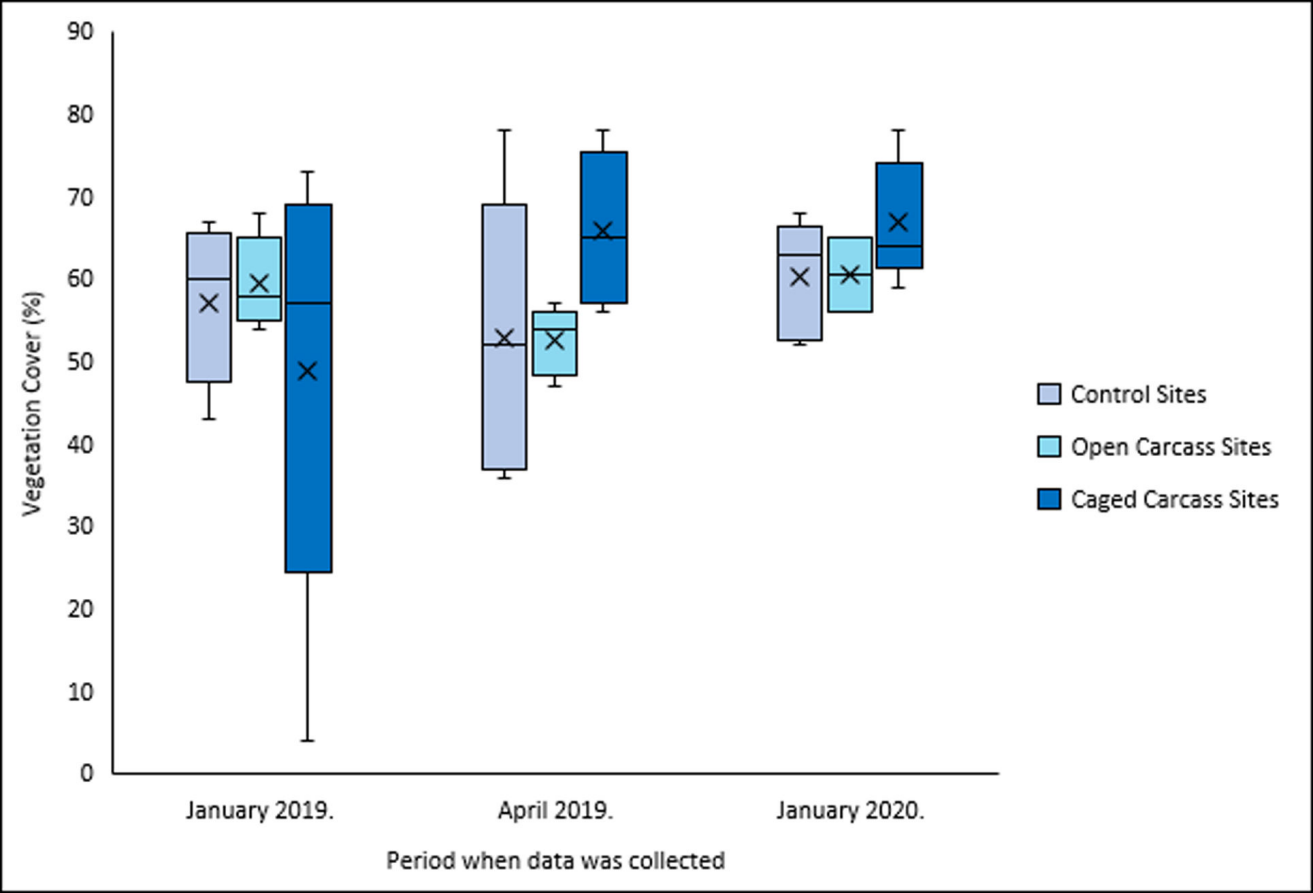
Vegetation cover for all survey sites for the three different carcass placement scenarios over the study period.

Vegetation cover patterns varied among treatments over the study period (figure 4). Control and open carcass sites showed minimal change (ending at 60% and 61%, respectively), while caged carcass sites demonstrated a notable increase from 49% to 67% (figure 4). However, none of these changes were statistically significant (table 2).

### 3.3. Plant biomass

**Figure 5.**
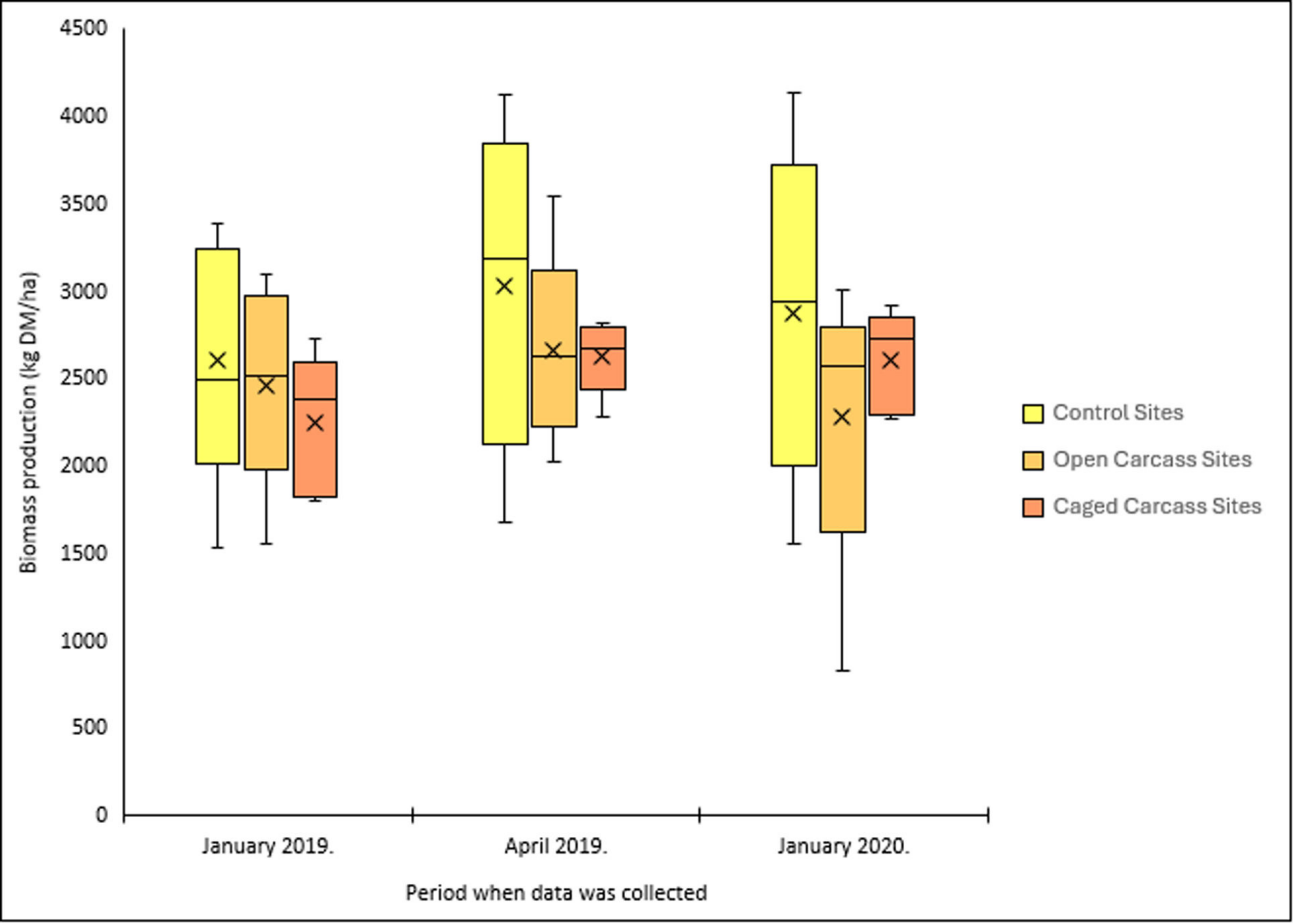
Variation in plant biomass for the three different carcass placement scenarios over the study period.

Plant biomass responses differed significantly among treatments (figure 5). Control sites showed a 10.6% increase (275 kg DM ha^−1^), while open carcass sites experienced a 7.2% decrease (176 kg DM ha^−1^). Caged carcass sites showed the greatest improvement with a 15.9% increase (317 kg DM ha^−1^).

### 3.4. Plant species richness and diversity

**Figure 6.**
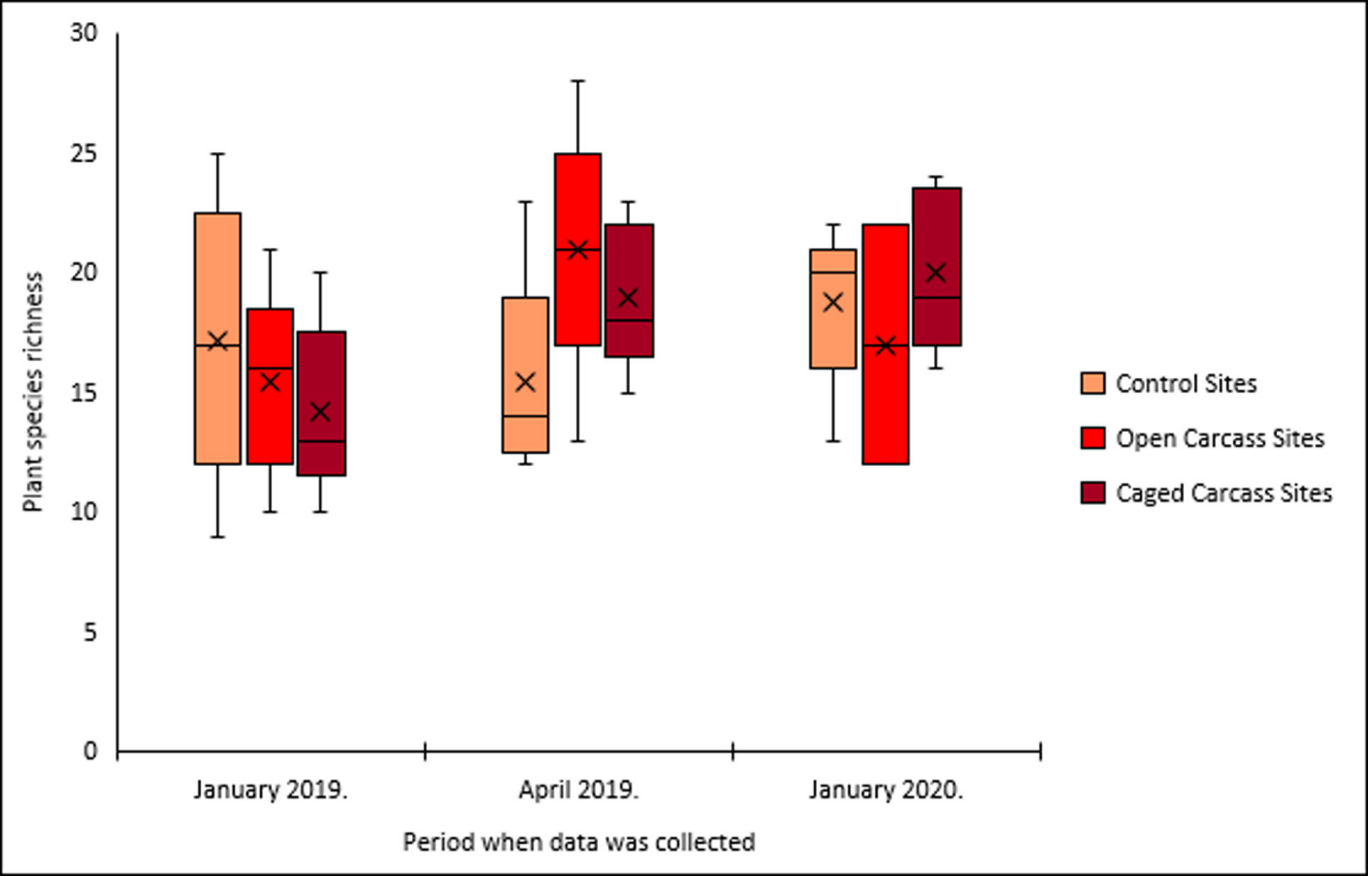
Variation in plant species richness for the three different carcass placement scenarios over the study period.

**Figure 7.**
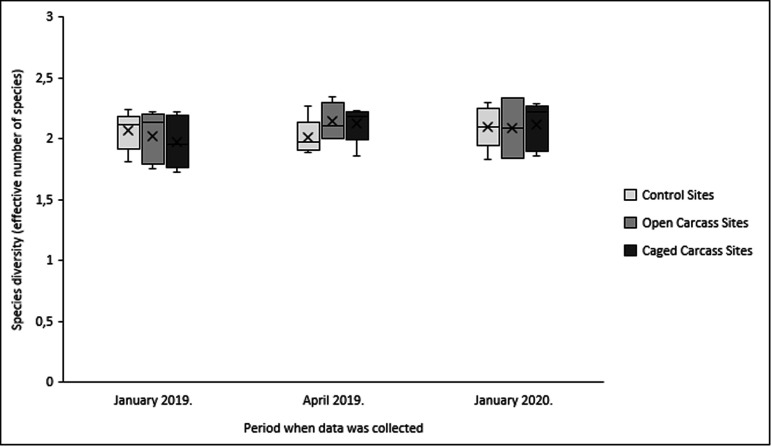
Variation in species diversity (effective number of species) for the three different carcass placement scenarios over the study period.

Species richness increased significantly at both carcass treatments but remained unchanged at control sites (figure 6). Open carcass sites gained two species, while caged carcass sites gained six species over the study period (electronic supplementary material, table S3). However, species diversity (effective numbers) remained constant at approximately two species across all treatments (figure 7).

### 3.5. Veld condition score

**Figure 8.**
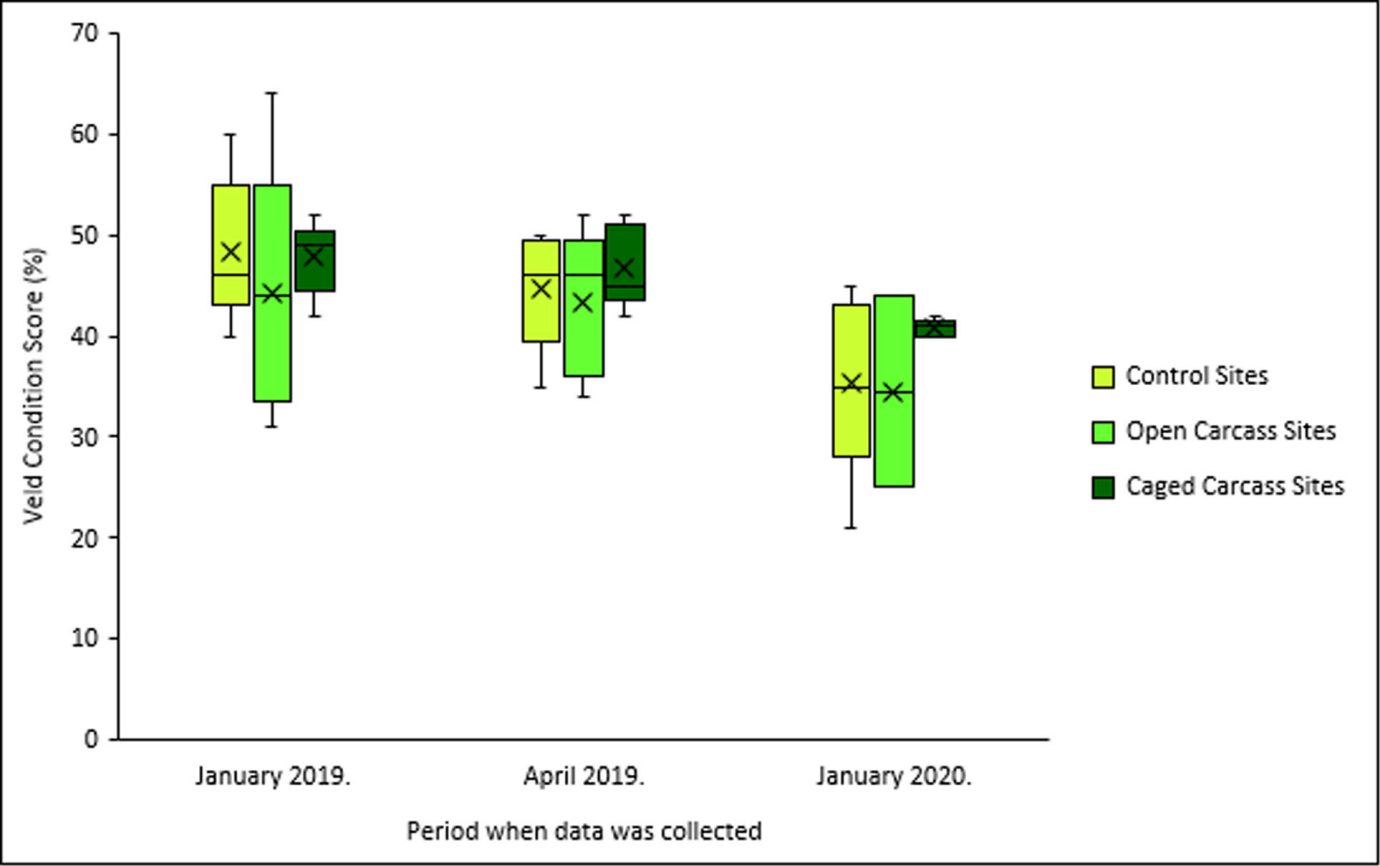
Variation in veld condition score percentage for the three different carcass placement scenarios over the study period.

All treatments showed declining VCS over time (figure 8). Control sites experienced the largest decrease (48% to 35%), while caged carcass sites showed the smallest decline (48% to 41%), indicating relatively better veld condition maintenance (table 2).

### 3.6. Results summary

See table 2.

## 4. Discussion

Our prediction that carcass decomposition would increase vegetation height was partially supported, with all treatments showing a 5 cm average increase over the 12 months period. However, contrary to our expectation that caged carcass sites would show greater increases due to concentrated nutrient inputs, both open and caged carcass sites exhibited similar height increases to control sites. These findings contrast with Bump *et al.* [13], who found that plant height increased at carcass sites compared with control sites, both for scavenged and intact carcasses, with this effect persisting for 2 years following European bison mortality due to elevated nitrogen levels in the soil. However, our results align with Collins [38] and Melis *et al.* [4], who also recorded no differences in vegetation height between carcass sites and control sites 1 year post-mortality. The lack of significant differences in our study may reflect the relatively short study duration, as some species require extended periods to express full growth responses to nutrient enrichment.

As predicted, caged carcass sites showed the greatest increase in vegetation cover (18% over 12 months), while open carcass sites showed minimal change (1% increase). However, the prediction that open sites would initially show decreased cover was not clearly demonstrated. The substantial cover increase at caged sites supports findings by Olsen [39], who documented increased vegetation cover along gradients from carcass centres outward, and aligns with Danell *et al.* [6], who found that herbivores avoided grazing near intact carcasses, leading to increased vegetation cover. Similarly, Bump *et al.* [13] found lower vegetation cover at open carcass sites due to scavengers trampling vegetation and dispersing the carcass around.

Biomass production results strongly supported our prediction that caged sites would show greater increases than open carcass sites. Caged carcass sites demonstrated a 15.9% biomass increase, while open carcass sites experienced a 7.2% decrease, probably due to scavenger trampling and carcass dispersal [5]. The significant biomass increase at control sites (10.6%) reflects normal seasonal growth patterns. Larger bare patches were created at the centre of caged carcass sites as the carcasses were not dragged or consumed by scavengers. Smaller patches were created at and around the open carcass sites where scavengers dragged and consumed some parts of the carcasses from the centre. Both the larger and smaller patches became an area of disturbance where there was an abundance of resources (such as high nutrient concentration and light) and reduced competition. A year later, the patches were recolonized by vegetation from the edges (as with [3]). Towne [5] found that 1 year after an ungulate died, biomass was lower in the centre compared with the area surrounding the carcass centre. Barton *et al.* [3] noted that recolonization of vegetation on the barren patches at the centre plots usually took place from the edge of the carcass site moving inwards. This means vegetation biomass increases in a gradient towards the centre of the carcass site, as recorded in our study.

Our prediction that both carcass treatments would exhibit higher species richness was fully supported. Caged carcass sites showed the most dramatic increase (plus six species), while open carcass sites and controls each gained two species. This finding aligns with a study done in south-central Sweden, where higher species richness was recorded at moose carcasses with minimal scavenging [39]. The higher species richness at caged carcass sites could be attributed to caged carcasses remaining in place and releasing more nutrients in a concentrated area along a gradient. In contrast, at open carcass sites, scavengers consume the meat and drag pieces away, resulting in fewer nutrients being released in the immediate vicinity of the carcass [5].

Contrary to our prediction of increased species diversity at carcass sites, effective species diversity remained constant across all treatments. This finding is consistent with Towne [5], who found that plant diversity changes require longer time periods to develop, with significant increases only occurring after 2 years due to pioneer species colonization. The lack of diversity change may also reflect the fact that carcasses do not disturb soil or expose buried seeds, limiting composition changes to species already present in the local seed bank [40].

Our prediction that VCS would improve at carcass sites was partially supported. While all treatments showed declining VCS over time, caged carcass sites experienced the smallest decrease (−7%) compared with control sites (−13%) and open carcass sites (−9%). This relative improvement suggests that decomposing carcasses create microhabitats that support the growth of Increaser 1 species (electronic supplementary material, table S3), thereby maintaining better veld condition. Increaser 1 species are moderately palatable and indicative of intermediate rangeland condition [41]. The wider dispersal of nutrients at open carcass sites, facilitated by scavenger activity, may explain why these sites showed intermediate VCS responses.

Our prediction that carcass effects would be most pronounced within a 2 m radius was supported by the observed patterns of biomass and plant cover changes. The creation of distinct nutrient gradients around carcass sites, with concentrated effects at caged carcass sites and more dispersed effects at open carcass sites, demonstrates how decomposition processes contribute to grassland heterogeneity. This spatial heterogeneity is crucial for maintaining ecosystem function and supporting diverse plant communities. Carcass decomposition creates lasting ecosystem changes that extend well beyond the initial decomposition period. Melis *et al.* [4] documented elevated soil nutrients and enhanced vegetation around European bison carcasses 5 years post-decomposition, while Danell *et al.* [6] observed fertile patches with increased plant growth 10 years after musk oxen deaths in the Canadian Arctic. These long-term effects generate ecosystem heterogeneity that 1-year studies cannot detect, suggesting that extended research periods are necessary to fully understand vegetation dynamics following decomposition. This natural process supports ecosystem health and resilience in grassland conservation areas like the Bankenveld.

An important limitation of this study is the relatively short time frame (two growing seasons), which makes it challenging to distinguish between immediate disturbance effects and longer-term nutrient enrichment effects. The initial vegetation response observed may primarily reflect disturbance by scavengers and the temporary absence of grazing pressure, rather than the enrichment effects of carcass decomposition. Future studies should extend observations over multiple growing seasons to allow vegetation communities sufficient time to recover from initial disturbance and to detect the full spectrum of nutrient cycling effects. This temporal consideration is crucial for understanding the true ecological impact of carcass decomposition in grassland ecosystems.

## 5. Conclusion

This study provides the first empirical evidence that large herbivore carcass decomposition significantly influences vegetation dynamics in southern African grasslands. The key finding is that carcass decomposition creates distinct microhabitats that enhance plant species richness and biomass production, with the magnitude of effects dependent on scavenger access. Caged carcasses, which concentrate nutrient inputs, promote greater vegetation responses than naturally scavenged carcasses, which disperse nutrients more widely (as with [12]).

These findings have important implications for grassland management and conservation. Carcass decomposition should be recognized as a natural process that contributes to ecosystem heterogeneity and supports biodiversity in Bankenveld grasslands. Rather than viewing animal mortality as a disturbance, managers should consider it as an integral component of ecosystem functioning that creates resource patches supporting diverse plant communities. This perspective is particularly relevant for conservation areas where natural scavenging processes should be maintained to optimize the ecological benefits of decomposition.

The study demonstrates that decomposition processes operate at multiple spatial and temporal scales, creating both immediate disturbance effects and long-term enrichment benefits that contribute to the structural and functional diversity of grassland ecosystems. Understanding these processes is essential for developing effective management strategies that maintain the ecological integrity of Bankenveld grasslands in the face of increasing anthropogenic pressures.

## Data Availability

Data can be accessed on Dryad [42]. Supplementary material is available online [43].
